# Quantifying Population-Level Risks Using an Individual-Based Model: Sea Otters, Harlequin Ducks, and the *Exxon Valdez* Oil Spill

**DOI:** 10.1002/ieam.1277

**Published:** 2012-01-03

**Authors:** Mark A Harwell, John H Gentile, Keith R Parker

**Affiliations:** † Harwell Gentile & Associates, LCPO Box 291267, Port Orange, Florida 32129-1267, USA; ‡ Harwell Gentile & Associates, LCBrewster, Massachusetts; § Data Analysis GroupCloverdale, California

**Keywords:** Ecological risk assessment, Population-level risks, Individual-based models, *Exxon Valdez* oil spill, Sea otters Seaducks

## Abstract

Ecological risk assessments need to advance beyond evaluating risks to individuals that are largely based on toxicity studies conducted on a few species under laboratory conditions, to assessing population-level risks to the environment, including considerations of variability and uncertainty. Two individual-based models (IBMs), recently developed to assess current risks to sea otters and seaducks in Prince William Sound more than 2 decades after the *Exxon Valdez* oil spill (EVOS), are used to explore population-level risks. In each case, the models had previously shown that there were essentially no remaining risks to individuals from polycyclic aromatic hydrocarbons (PAHs) derived from the EVOS. New sensitivity analyses are reported here in which hypothetical environmental exposures to PAHs were heuristically increased until assimilated doses reached toxicity reference values (TRVs) derived at the no-observed-adverse-effects and lowest-observed-adverse-effects levels (NOAEL and LOAEL, respectively). For the sea otters, this was accomplished by artificially increasing the number of sea otter pits that would intersect remaining patches of subsurface oil residues by orders of magnitude over actual estimated rates. Similarly, in the seaduck assessment, the PAH concentrations in the constituents of diet, sediments, and seawater were increased in proportion to their relative contributions to the assimilated doses by orders of magnitude over measured environmental concentrations, to reach the NOAEL and LOAEL thresholds. The stochastic IBMs simulated millions of individuals. From these outputs, frequency distributions were derived of assimilated doses for populations of 500 000 sea otters or seaducks in each of 7 or 8 classes, respectively. Doses to several selected quantiles were analyzed, ranging from the 1-in-1000th most-exposed individuals (99.9% quantile) to the median-exposed individuals (50% quantile). The resulting families of quantile curves provide the basis for characterizing the environmental thresholds below which no population-level effects could be detected and above which population-level effects would be expected to become manifest. This approach provides risk managers an enhanced understanding of the risks to populations under various conditions and assumptions, whether under hypothetically increased exposure regimes, as demonstrated here, or in situations in which actual exposures are near toxic effects levels. This study shows that individual-based models are especially amenable and appropriate for conducting population-level risk assessments, and that they can readily be used to answer questions about the risks to individuals and populations across a variety of exposure conditions. Integr Environ Assess Manag 2012; 8: 503–522. © 2012 SETAC

## INTRODUCTION

Ecological risks and ecological recovery following environmental disturbances are critical issues in understanding and managing ecosystems. Among other issues, they are central to determining if an ecosystem is vulnerable to a particular stressor, evaluating the ability of alternate management actions to achieve environmental goals, designing an ecological restoration plan, evaluating the efficacy of risk-reduction measures such as remediation, assessing observed ecological responses to evaluate the necessity for adaptive management, and assessing when, and if, an ecological system has recovered from a past disturbance. Consequently, there is a broad-based need for appropriate, effective, and scientifically defensible tools for assessing ecosystem risks and recovery for managing human effects on the environment. One critical component of that need is the ability to assess risks to populations quantitatively.

We have argued elsewhere (Gentile and Harwell 2001; Gentile et al. 2001; Harwell 1997; Harwell et al. 1996; Harwell, Gentile, Cummins et al. 2010; Harwell and Gentile 2000) that assessing the condition and recovery of ecological systems in the presence of multiple natural and anthropogenic stressors requires a hierarchical approach, fundamentally based on the ecological risk assessment framework (USEPA 1992, 1998), focused on carefully selected ecological endpoints or valued ecosystem components (VECs), and cognizant of natural variability.

In Harwell and Gentile (2006), we applied these concepts to assessing the significance of any continuing effects on the Prince William Sound (PWS), Alaska, ecosystem more than 15 years after the *Exxon Valdez* oil spill (EVOS) (see also Harwell, Gentile, Cummins et al. 2010). In the ecological risk context, the issue is 2-fold, 1 pertaining to exposure and the other to effects: 1) Have the stressors caused by the oil spill decreased to the point where no further risk exists to the PWS ecosystem and its biota, and 2) Have VECs in PWS that were initially impacted now recovered?

The EVOS occurred on 24 March 1989, with over 250 000 barrels of Alaska North Slope crude oil released into northeastern PWS (Galt et al. 1991; Harrison 1991; NOAA 1992; Wolfe et al. 1994; Spies et al. 1996). Neff et al. (1995) reported that 782 km of the PWS shoreline (approximately 16%) was oiled to some degree after the spill. Four stressors resulted from EVOS: 1) volatile organic compounds (VOCs), which dissipated quickly but posed an early inhalation risk to some species, such as killer whales, 2) oiling, which caused loss of thermoregulation in the cold PWS waters and thus probably caused the majority of observed mortality to seabirds and sea otters, 3) polycyclic aromatic hydrocarbons (PAHs), which pose a longer-term toxicological risk to exposed organisms, and 4) the set of stressors caused by the cleanup activities of intertidal and shoreline habitat, including physical disturbance from high-pressure high-temperature water, extensive human presence, noise, water pollution, and wildlife disruption.

PWS is a very dynamic system, with extreme storms and high-energy wave and tidal regimes; consequently, residual *Exxon Valdez* oil (EVO) was largely eliminated naturally from shorelines in the initial months-to-few years after the spill, assisted by the massive cleanup operation during the summers of 1989–1991 (Teal 1991; Harrison 1991; Mearns 1996). By 2001 only approximately 0.1%–0.3% of the initial spill volume remained (Short et al. 2004), primarily in small, widely dispersed pockets of subsurface oil residues (SSOR) located in the mid-to-upper intertidal zone under a 15–25 cm-thick layer of clean sediments (Hayes and Michel 1999; Hayes et al. 2010), which in turn were located under a surface covering of stable armor composed of coarse gravel, cobble, and boulders (Hayes and Michel 1999; Michel et al. 2006; Taylor and Reimer 2008; Hayes et al. 2010). SSOR rarely occurred in unarmored, finer-grained sediments. Consequently, these subsurface deposits are released only very rarely and episodically into the coastal environment (Boehm et al. 2004; Neff et al. 2006) (otherwise, the residues would long since have left the system). For example, SSOR could be released by physical disturbance of the subsurface sediments during extreme storms, but after 2 decades, virtually all such storm-induced releases have already occurred. Similarly, SSOR could be released via bioturbation by sea otters' excavating pits when harvesting infauna; however, whereas this may have been a release mechanism in the early years after the oil spill, at present such SSOR excavations by sea otters occur only rarely (see the quantitative risk assessment in Harwell, Gentile, Johnson et al. [2010]).

In addition to the physical release of SSOR, PAHs in the SSOR may gradually be released into the aqueous phase of the pore-water within the sediments and then transported to the surface water. As shown by Pope et al. (2011), however, such releases are at extremely low levels, limited by: 1) the very low solubility of remaining PAHs and the controlling equilibrium partitioning dynamics, 2) the very low permeability of the fine-grained sediments in which SSOR persist, and 3) the tidally driven, very large volumes of water that flow through the higher-permeability surface sediments, which greatly dilute any dissolved PAHs leached from subsurface deposits. As a result, these SSOR-derived PAHs in seawater are indistinguishable from background. However, in surface sediments, there are PAHs sorbed onto sediment particles that may become bioavailable if ingested by sediment-processors or filter-feeders (NRC 2003), including PAHs from pyrogenic sources, PAHs from other petrogenic sources, PAHs from biogenic sources, and, in some areas, low levels of PAHs that derived from EVO.

As a result, 3 of 4 classes of stressors resulting from EVOS, VOCs, oiling, and cleanup activities, were eliminated from the system many years ago. Presently the sole risk to PWS biota is the potential exposure to residual EVOS-derived PAHs, located either in the SSOR deposits or in the background levels of potentially bioavailable PAHs in EVOS-oiled areas.

Using the Gentile and Harwell (1998) criteria for assessing ecological significance (see also Harwell et al. 1994), Harwell and Gentile (2006) evaluated a suite of approximately 2 dozen VECs that characterize the PWS ecosystem, concluding that by that point in time recovery had essentially occurred for almost all of the VECs; similar conclusions were presented in Integral Consulting (2006), with a few exceptions. However, the *Exxon Valdez* Oil Spill Trustees (EVOSTC 2010) continued to report several endpoints as not recovered, including characterizing sea otters and Harlequin Ducks as “recovering” but not yet recovered species.

The sea otter (*Enhydra lutris*) is found in Alaskan coastal waters, feeding on benthic invertebrate epifauna and infauna, including clams, mussels, sea urchins, snails, and crabs (Bodkin and Ballachey 1997). Thus, its foraging habitat is limited in PWS to the relatively narrow zone along the shorelines that is sufficiently shallow for sea otters to reach the benthic communities (Bodkin and Ballachey 1997). At the time of EVOS, the PWS population of sea otters was rapidly expanding and increasing, as it was becoming reestablished throughout the northern Pacific after near-extinction by 1911, when the International Fur Seal Treaty ended harvesting (Doroff et al. 2003); for example, Estes (1990) reported the SE Alaska sea otter population increased at an annual rate of 17.6% from 1975 to 1987.

Their dense fur and unusually high metabolic rate make sea otters particularly vulnerable to oiling, both from decreased buoyancy and from hypothermia with loss of insulating capacity (Lipscomb et al. 1994), which is especially important because PWS is at the northern limit of the population range (Johnson and Garshelis 1995). Sea otters are also vulnerable to inhalation of toxic VOCs or ingestion of oil-contaminated food, exacerbated by increased rates of grooming after oiling (Johnson and Garshelis 1995). A total of 871 carcasses were found after EVOS, and 123 additional sea otters died in rehabilitation centers, totaling an observed mortality of approximately 1000 (Estes 1990; Loughlin et al. 1996; EVOSTC 2002). Garrott et al. (1993) estimated total sea otter mortality from EVOS in PWS at 2800. Johnson and Garshelis (1995) noted that by 1991 the sea otter population in the oiled areas equaled or surpassed prespill levels (based on 1984 estimates) at all their survey sites. Bodkin and Ballachey (1997) and Bodkin and Dean (2000) reported PWS sea otter abundance of 12 000–13 000 as surveyed in 1994, 1995, and 1999, exceeding the prespill estimates of 5000–10 000 (Burn 1994).

Despite an approximate 4% annual increase of the postspill sea otter population in PWS as a whole, an issue has been raised concerning the subpopulation of sea otters at Northern Knight Island (NKI). For instance, EVOSTC (2010) stated that although there had been a slow increase in the sea otter population at NKI since 2005, there had been a greater rate of overall increase in the PWS population as a whole, and therefore considered sea otters to be recovering but not yet recovered. Bodkin et al. (2002) and Dean et al. (2002) attributed the effects potentially to SSOR (no current effects are posited for the sea otters elsewhere in PWS, even though SSOR is not limited to NKI). Short et al. (2006) suggested that, when digging in sediments for food, NKI sea otters and seaducks may encounter SSOR in sufficient frequency and quantity to affect their health. In judging the plausibility of this suggestion, the issue, then, becomes one of quantitatively assessing the current risks from SSOR to NKI sea otters.

Similarly, the potential for long-term effects on the Harlequin Duck (*Histrionicus histrionicus*) population is the subject of continuing discussion. These are small seaducks that inhabit shallow marine intertidal zones off rocky shorelines during winter (Robertson and Goudie 1999). An estimated 500–1000 Harlequin Ducks (approximately 3%–7% of the wintering population) were killed as a result of direct exposure to EVO during and immediately after the spill (Esler et al. 2002; Rosenberg et al. 2005). Within weeks of the oil spill, concerns were expressed about potential persistent adverse toxic effects on Harlequin Ducks from ingesting oiled prey, especially mussels (Patten et al. 2000). Some studies conducted several years after EVOS have reported decreased abundance, attributing it to: 1) reduced overwintering survival of females and/or unsuccessful reproduction caused by EVOS (Esler et al. 2002; Patten et al. 2000), 2) degraded habitat quality not associated with EVOS (Day et al. 1995, 1997; Irons et al. 2000; Wiens et al. 2004), or 3) exposure to hydrocarbons and, by inference, continuing toxic effects from EVOS, as indicated by elevated cytochrome P4501A [CYP1A] activity (Trust et al. 2000; Esler et al. 2002; Esler 2008; Esler and Iverson 2010). Based on a weight-of-evidence evaluation from several studies, Wiens et al. (2010) concluded no indication of continuing population-level impacts, in terms of either abundance or demographics, on Harlequin Ducks from EVOS. On the other hand, EVOSTC (2010) concluded that there continues to be a risk of exposure to hydrocarbons from EVOS 2 decades after the oil spill, based in part on the CYP1A data (see also Esler 2008). Consequently, EVOSTC (2010) classifies Harlequin Ducks as “recovering but not recovered,” based on the recovery objective that “…biochemical indicators of hydrocarbon exposure in harlequins in oiled areas of PWS are similar to those in harlequins in unoiled areas.” We have argued elsewhere (Harwell and Gentile 2006; Harwell et al. 2012) that biochemical markers are inappropriate to use as recovery objectives, because biomarkers only can indicate exposure, not effects, and recovery by definition is an effects issue. Nevertheless, the relevant issue here becomes one of quantitatively assessing the current risks from EVOS to PWS seaducks to answer the question, is it possible or not for the residual PAHs from EVOS to cause effects on seaducks?

To assess the possibility that residual PAHs from EVOS could continue to cause adverse effects on those 2 species, Harwell, Gentile, Johnson et al. (2010) and Harwell et al. (2012) conducted quantitative ecological risk assessments (ERA) on PWS sea otters and seaducks, respectively. In each case, those authors developed a conceptual model of all plausible pathways of PAH exposures to the animals; converted the conceptual models into quantitative, stochastic, individual-based models (IBMs), parameterized with empirical data on those species as well as data on the SSOR and other EVOS-derived PAHs in PWS; developed chronic toxicity reference values (TRVs) for PAHs appropriate for sea otters and seaducks, based on the best-available applicable data in the literature; simulated millions of sea otters and seaducks to derive assimilated doses of PAHs; and assessed the frequency distributions of exposures and resulting effects to reach conclusions on the magnitude of the potential risks to individual sea otters or seaducks.

In both studies, the authors assessed the chronic doses to the 99.9% quantile individuals (i.e., the 1-in-1000th most-exposed individual sea otters or seaducks). This approach, coupled with several other conservative attributes built into the models, provides a very conservative estimate of risks. The results indicated that the assimilated doses for sea otters were approximately 30–125 times lower than the no-observed-adverse-effects-level (NOAEL) TRV threshold, and approximately 75–310 times lower than the lowest-observed-adverse-effects-level (LOAEL) TRV threshold. For the Harlequin Ducks, the assimilated doses were approximately 400–4000 times lower than the NOAEL and LOAEL TRVs, respectively. Based on these results, the authors concluded that there was essentially no remaining individual-level risk to sea otters or seaducks from EVOS (Harwell, Gentile, Johnson et al. 2010; Harwell et al. 2012).

In the present study, we extend the use of the individual-based models to examine the potential for population-level effects. Under present PAH exposures, no individual-level effects are plausible and thus no population-level effects could occur. However, in a hypothetical environment in which the PAH concentrations are heuristically increased to attain TRV levels, the IBM-class of models provides a useful tool to assess population-level risks, a tool that would equally be useful for situations in which actual exposures are close to toxicity threshold values. The stochastic IBMs allow the generation of frequency distributions that represent the range of possible effects on individuals by capturing the underlying variability in exposures. These frequency distributions can be used to project the environmental exposures that would be necessary to cause detectable effects in populations. We believe the approach used here would be generally applicable to using stochastic individual-based models to quantitatively assess population-level risks.

## INDIVIDUAL-BASED MODELS

### IBMs and ecological risk assessments

Individual-based models have been used for several decades as alternatives to more traditional analytical population models (DeAngelis and Gross 1992; DeAngelis and Mooij 2005; see also Barnthouse [2007] and Munns et al. [2008] for overview of IBMs and other population-level models). The concept of IBMs for ecological models is that, instead of directly modeling population-level attributes (e.g., birth and death rates, predation–prey interactions) as solutions of differential or difference equations, IBMs simulate a large number of individual organisms, parameterized for specific individual-level attributes; population-level characteristics collectively emerge from the results. IBMs have been used for a wide range of species, from modeling of forest trees (Botkin et al. 1972; Shugart 1984; Urban and Shugart 1992; Liu and Ashton 1995) to birds (Fleming et al. 1994; Wolff 1994), fish (Gaff et al. 2000), and even earthworms and spiders (Baveco and De Roos 1996; Topping 1999). In a conceptual sense, the rationale for IBMs is to mimic the real-world situation, i.e., to reflect in a model the emergent properties that are characteristic of actual hierarchical systems such as ecosystems (von Bertalanffy 1968). For example, how a population of sea otters responds to a stressor in the environment only emerges through the collective responses of the individual sea otters within the population rather than through a process acting on the population as an entity, and the IBM merely seeks to reflect that relationship.

The IBM approach became feasible when digital computing capabilities were sufficient to handle each of the actions of a large number of individuals. An important advantage of IBMs is the ability to readily incorporate into the models alternative behaviors, spatial and temporal heterogeneity, thresholds of actions or processes, activities in which an individual chooses among alternatives, and other nonlinear processes, none of which can be readily addressed in models developed primarily for their ability to produce analytical solutions. Indeed, in the more traditional class of population models (e.g., age-class and other matrix-based models, differential- or difference-equation-based models, among many others) (Munns et al. 2008; Schmolke et al. 2010), many of the most important aspects of population-level characterization had to be ignored, linearized, assumed away, or otherwise not explicitly addressed. In contrast, an IBM, if adequately parameterized, can directly and realistically simulate essentially without constraint the activities, processes, and environmental conditions as experienced by individual organisms. So, for example, when attempting to simulate complex and often probabilistic toxicological risk pathways of an ecological risk assessment, an IBM can explicitly present differing exposure regimes from 1 individual to another, or 1 day to another, just as an individual organism might experience the natural heterogeneity of exposures encountered in the real world. Consequently, the IBM class of models is ideal for assessing toxicological risks to individuals and populations, such as the sea otters and seaducks in this study. To this point, IBMs have not been used widely in ecological risk assessments (Forbes et al. 2010; Schmolke et al. 2010; see also Munns et al. [2008] for discussion), but we believe they have considerable potential for broader application in ecological risk assessments and other applications, as discussed below. Note that the approach presented here does not use IBMs as population models per se, i.e., we do not model population abundance or population processes such as fecundity, mortality, age-class structure, immigration and emigration, competition, compensatory responses. Rather, we model the direct lethal and population-relevant sublethal effects from chronic exposures to toxic PAHs. This allows us to show how the population might change in relative abundance, thereby quantitatively assessing whether or not a significant risk exists. Two particular advantages of this approach are: 1) a greatly reduced demand for data, especially for many difficult-to-quantify population dynamics parameters, and 2) a much more direct characterization of the exposures and risks experienced by the animals as they occur in the actual environment.

### Sea otter individual-based model

**Figure 1:**
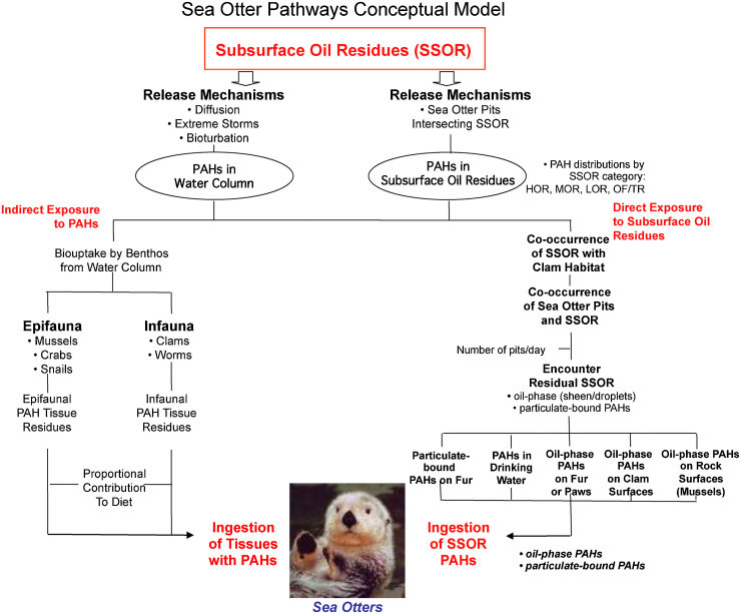
Sea otter exposure pathways conceptual model. Exposure pathways conceptual model for sea otters at Northern Knight Island from PAHs in subsurface oil residues (SSOR), showing the release mechanism of direct exposure to PAHs by intersection of SSOR by a sea otter pit, including ingestion of both oil-phase and particulate-bound PAHs, and the release mechanisms of indirect direct exposure to PAHs, through ingestion of PAHs in the diet. Reprinted by permission of Taylor & Francis (http://www.tandf.co.uk/journals) from Harwell, Gentile, Johnson et al. (2010).

Harwell, Gentile, Johnson et al. (2010) assessed the current risks to sea otters in Prince William Sound associated with EVO residues. A conceptual model (Figure 1) that presents all plausible pathways for PAH exposures, whether from EVOS-derived sources or not, was prepared and subsequently converted into a quantitative individual-based model based on empirical data. The IBM was developed using the simulation language *Stella*™ (ver. 9.1.2; © ISEE Systems [http://www.iseesystems.com]) to quantify assimilated doses of PAHs from each exposure pathway to sea otters located in areas of NKI that were initially oiled and to sea otters in reference (unoiled) areas.

An important factor in modeling assimilated doses of PAHs is that they are readily metabolized and consequently do not biomagnify through the food web (Eisler 1987; Neff 2002). This was demonstrated in empirical studies in which all PAH concentrations decreased when moving up a simple marine food web (Broman 1990; Broman et al. 1990). This contrasts with slowly metabolized polychlorinated biphenyls (PCBs), which significantly bioaccumulate and therefore present a serious trophic biomagnification risk, as seen in high levels of PCBs in apex predators like killer whales (Ross et al. 2000). Because of the absence of biomagnification, the sea otter IBM could accurately simulate assimilated doses of PAHs on a daily basis (e.g., there is no day-to-day carryover of effects), permitting the quantification of chronic average daily assimilated doses to a population of sea otters.

In this model, the dominant exposure pathway involves a sea otter foraging for infauna in the intertidal zone and digging a pit that directly intersects a patch of SSOR. Because unoiled areas have no SSOR, reference sea otters could not be exposed via this pathway. The other non-SSOR-intersecting pathways for sea otters in both oiled or unoiled areas were also modeled using existing PAH concentrations in their respective areas.

Four factors affect the probability of a sea otter digging a pit that intersects SSOR: 1) the distribution of SSOR patches across tidal zones and shoreline subdivisions, 2) the distribution of clam habitat across tidal zones, 3) the co-occurrence of clam habitat and SSOR, and 4) the number of pits excavated per sea otter per day in the intertidal zone (which varies by age and gender). The probability of a co-occurrence of a sea otter pit and SSOR was modeled implicitly, rather than explicitly, as follows. For each individual sea otter during each time step (1-h increment), the model randomly assigns these choices:

- Whether or not the sea otter fed during that time step
- If so, whether the foraging occurred in the intertidal zone (ITZ) or the subtidal zone (STZ)
- If in the ITZ, whether the foraging was for epibenthos or infauna (i.e., excavating a pit to collect clams)
- If foraging for infauna, then choosing the size of the sea otter excavation pit
- If a pit was excavated, then choosing the location of the pit with respect to the distribution of the SSOR (i.e., if an intersection of SSOR occurred or not)
- If SSOR was intersected, then choosing the nature of the SSOR (among the categories of high oil residues [HOR], medium oil residues [MOR], low oil residues [LOR], oil-film traces [OF], or no visible oil residues [NONE])
- For the specific SSOR category selected, selecting the total PAH (TPAH) concentration for that particular pit, sampled from the lognormal distribution of TPAHs measured for that category of SSOR
- Choosing the specific distribution of the 40 modeled PAH analytes within the TPAH, again randomly sampled from the distribution of PAH proportions measured for that category of SSOR.

Thus, by the end of a particular time step, if a sea otter dug a pit that intersected SSOR, then the model assigned the quantity of SSOR-derived PAHs for each of 40 analytes, and that quantity was subsequently allocated among the various exposure and assimilation pathways to the sea otter (Figure 1). These exposure pathways distinguished oil-phase PAHs (e.g., direct contact with actual SSOR) from particulate-phase PAHs (e.g., PAHs sorbed onto the plume of sediment particulates generated by the digging process). Oil-phase PAHs could be ingested and completely assimilated via several pathways, including consumption of SSOR on the surfaces of clams or mussels, SSOR that adhered to the sea otter's paws while digging, etc.

If no SSOR intersection occurred during the time step (whether because the sea otter did not feed in that hour, feeding occurred in the STZ, feeding occurred in the ITZ but for epifauna, or feeding occurred in the ITZ for infauna but the pit did not intersect SSOR), then a sea otter in an initially oiled area still assimilated PAHs from prey, sediments, and seawater at the concentrations that currently exist in the respective environmental compartments. To quantify the assimilated doses to unoiled sea otters, the SSOR-related pathways were bypassed in the model, but all other exposure pathways were simulated using environmental data from unoiled areas.

Each of the SSOR-related options in the co-occurrence simulations was based on model parameterization using the most recent available empirical data on: 1) the locations and spatial distributions of SSOR in the ITZ, 2) the frequency distributions of the SSOR categories (e.g., HOR, MOR), 3) the SSOR category-specific variability of TPAH, and 4) the SSOR category-specific proportional distributions of the PAH analytes. These parameterizations were based on the SSOR surveys conducted by NOAA in NKI in 2001 and 2003 (Short et al. 2004, 2006) and on PAH chemical analyses carried out on SSOR samples collected in 2002, as detailed in Harwell, Gentile, Johnson et al. (2010).

The sea otter feeding behavior and related sea otter data (e.g., feeding time periods, frequency of dives in ITZ versus STZ, frequency of ITZ dives foraging for infauna, frequency distribution of infaunal dietary species, number of pits per feeding hour) for both oiled and reference sea otters were derived from the observational database of over 3000 sea otter dives developed by Garshelis and Johnson (reported in Harwell, Gentile, Johnson et al. [2010]) for 7 age and gender classes of PWS sea otters. Other data on sea otter characteristics were derived from the literature, including sea otter energetics (Dean et al. 2002), sea otter body mass (Dean et al. 2002; Rotterman and Monnett 2002; Ballachey et al. 2003), surface area of sea otter fur (Kenyon 1969; Davis et al. 1988), paw size (Tarasloff 1973), and other model parameters (e.g., energy density of each prey category [Dean et al. 2002]). The PAH concentrations and distributions of prey, sediments, and seawater were derived from chemical analyses conducted on samples taken in oiled and unoiled areas (Boehm et al. 2004, 2007; Neff et al. 2006; Brannon et al. 2007). The sources of the data used in the sea otter model were described and cited in Harwell, Gentile, Johnson et al. (2010).

There is considerable variability in PAH exposures to PWS sea otters because of differing concentrations and relative proportions of each PAH analyte in each pathway that leads to exposures. To capture that exposure variability, the model simulated 1000 y of daily assimilated doses by randomly and separately assigning in each simulation hour and within each day, a specific value for each stochastic parameter, sampled from the distributions discussed above. From this distribution of daily doses, the chronic average daily doses were derived for each individual in a simulated population of sea otters. Note that the number of individuals simulated in the population (500 000) was chosen to reduce the simulation coefficient of variability to below 0.1% as detailed in Harwell, Gentile, Johnson et al. (2010). In this way, the simulated population captured the full range of plausible exposures to PAHs that could occur to sea otters at NKI (actual subpopulation size on order of 75 individuals). Because this IBM predicts toxicological risks, not population abundance or densities, there are no impediments to projecting any number of members of a hypothetical population (e.g., there are no issues of limits such as “carrying capacity”). However, in an actual population that experiences significant reduction in numbers, other processes not modeled here, such as compensatory mechanisms, might become important, thereby potentially reducing adverse effects (meaning our risk estimates are conservative).

It should also be noted that the IBM was limited to older pup, juvenile, and adult sea otters. We did not model the younger pup class because these individuals only feed on maternal milk, which does not concentrate PAHs, so the weight-adjusted assimilated doses would be no greater than for the adult female with pups (and likely less because PAHs are not completely transferred into milk). Also, embryonic sea otters were not simulated, as there are no applicable toxicity data and no data to parameterize the transfer of PAHs transporting the placenta. However, because there is no evidence of reduced reproduction or fecundity, as characterized by the ratio of the number of pups to the total number of juveniles + adults, even in sea otters within the spill zone soon after the oil spill (Garshelis and Johnson 2001; Dean et al. 2002), let alone more than 20 years later, there is no indication of increased embryonic mortality or reduced embryonic survival rates. Similarly, the IBM did not model sea otter behavior; so, for example, if there were avoidance of oil, the model would not reflect that (thereby providing a conservative estimate of risk). However, as discussed in more detail in Harwell, Gentile, Johnson et al. (2010), we believe that the conceptual model shown in Figure 1 captures all of the plausible pathways of toxicological exposure to the classes of sea otters modeled, and each of the pathways in the conceptual model was quantified in the IBM.

To provide highly conservative estimates of potential effects from the exposures, Harwell, Gentile, Johnson et al. (2010) rank-ordered assimilated doses and emphasized the 99.9% quantile sea otters (i.e., 1-in-1000 most-exposed individuals) for the effects assessment; however, values for other quantiles were also calculated, providing the bases for the inferences concerning population-level effects discussed below. A suite of sensitivity analyses explored the effects of alternative model parameters and model structures on the results. Altogether >1 billion sea otter-hours were simulated to capture the environmental, SSOR, and sea otter variability, demonstrating the exceptional ability of a well-designed IBM to thoroughly explore variability and uncertainty in exposures. Further details of the model and its parameterization are provided in Harwell, Gentile, Johnson et al. (2010).

The effects component of the ecological risk assessment used a standard US Environmental Protection Agency (USEPA)-approved approach to establish the TRVs for PAH exposures to sea otters (USEPA 2007). The TRV is defined as the dose from chronic exposures above which ecologically relevant effects might occur to wildlife species and below which it is reasonably expected that such effects would not occur (USEPA 2005). The ratio of the model-simulated dose to the TRV value is the hazard quotient (HQ), where an HQ ≥ 1 indicates the exposure could lead to chronic effects and HQ < 1 indicates no ecologically relevant adverse chronic effect would occur. The TRVs used in this study were derived from USEPA-approved set of experimental data in its Eco-SSL database (USEPA 2007). To develop that database, USEPA screened several thousand dose-response toxicity experiments on PAHs and selected approximately 40 studies that met all criteria for data quality and applicability to mammalian wildlife. Harwell, Gentile, Johnson et al. (2010) extracted the mammal-relevant NOAEL and LOAEL values from each EPA-approved toxicity study, based on the population-relevant endpoints of mortality, growth, or reproductive effects. Following the USEPA-approved protocol of Sample et al. (1996), the ingestion dose data (based primarily on rat or mouse experiments, as is typical for mammalian toxicology) were normalized on a body-weight basis to apply to sea otters. Using these data across all tests and species, the geometric means and standard deviations were generated, from which the geometric 95% lower confidence limits (CL) were calculated for use as the TRVs. Geometric statistics are used in USEPA guidance as better representing distribution of toxicity data across experiments and species. The geometric 95% lower CL is often used in ecological risk assessments to address interspecies sensitivity differences for untested species (see for instance the USEPA-issued guidance for establishing water quality criteria [Stephan et al. 1985] and sediment quality criteria [USEPA 1993]), rather than applying safety factors as is commonly done in human health risk assessments. Hence, using this approach to setting the TRVs here accommodates the uncertainty associated with using dose-response data based on laboratory experiments on rodents to estimate effects on a marine mammal. Following USEPA (2007), TRVs were separately established for the lower-molecular-weight PAHs (i.e., PAHs with 2- and 3-ring structures) and the higher-molecular-weight PAHs (i.e., having 4-, 5-, or 6-ring structures), as well as for total PAHs, allowing comparison to the literature, which is often reported as TPAH.

Model outputs for the 99.9% quantile individuals in each sea otter class were compared against the appropriate TRVs. The resulting HQ for the 99.9% quantile sea otters based on 4–6-ring PAHs were 0.0081–0.034 for the no-observed-adverse-effects-level (NOAEL), or approximately 30–125 times lower than the TRV threshold, and 0.0032–0.0133 for the lowest-observed-adverse-effects-level (LOAEL), or approximately 75–310 times lower than the TRV threshold. Even lower NOAEL and LOAEL HQs (NHQs and LHQs, respectively) resulted for 2–3-ring PAHs and TPAH. A series of sensitivity analyses showed these results were robust, and the authors concluded that there is currently no plausible risk to any individual sea otter from SSOR at NKI (Harwell, Gentile, Johnson et al. 2010).

### Harlequin Duck individual-based model

**Figure 2:**
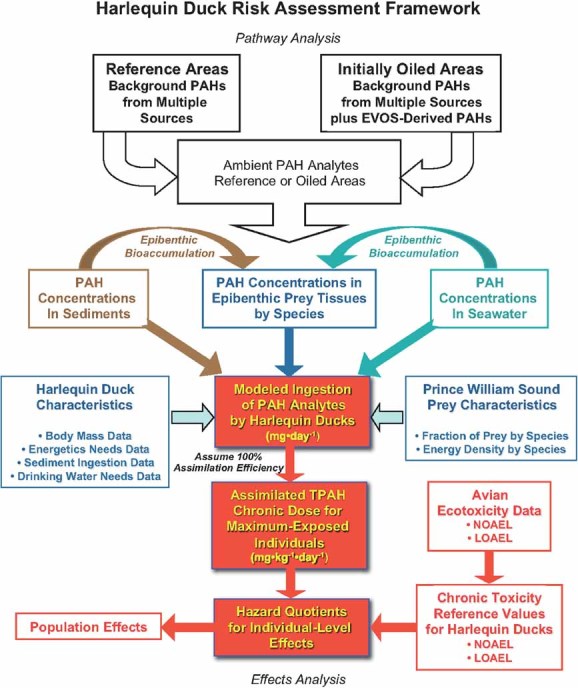
Harlequin Duck risk assessment framework. Pathways conceptual model showing: 1) the sources of PAHs in the PWS environment in reference and initially oiled sites, 2) concentrations of PAHs in sediments, seawater, and prey tissues, 3) risk assessment model inputs, including characteristics of Harlequin Ducks in PWS and their prey, 4) elements of the risk assessment model, 5) generation of assimilated doses, 6) development of chronic toxicity reference values for Harlequin Ducks, and 7) assessment of individual- and population-level effects. Reprinted by forthcoming permission of Taylor & Francis (http://www.tandf.co.uk/journals) from Harwell et al. (2012).

Harwell et al. (2012) assessed the current risks associated with EVOS to Harlequin Ducks in PWS, quantifying assimilated doses of PAHs from all plausible sources and exposure pathways to seaducks in PWS in oiled and unoiled areas. Again, the simulation language *Stella*™ was used to quantitatively assess the toxicological risks to 8 classes of seaducks, adult and juvenile males and females in summer and winter (note that we did not simulate risks to seaduck embryos or hatchlings). A conceptual model was developed describing the potential pathways of PAH exposures, including from PAHs derived from residual EVO (Figure 2). In developing this conceptual model, 5 potential exposure pathways were considered: 1) direct encounter with surface oil residues (SOR), 2) direct encounter with subsurface oil residues (SSOR), 3) consumption of prey tissues containing PAHs, 4) consumption of sediments containing PAHs, and 5) consumption of seawater containing PAHs. No other pathways of environmental PAH risks to adult or juvenile seaducks were considered to be plausible.

Taylor and Reimer (2008) reported that by 2002 PWS had very little or no SOR (covering <0.2% of the total surface area of surveyed sites). The SOR that did exist had weathered to patches of hard, asphalt “pavement” or highly weathered tar splats, located primarily in middle to upper intertidal zones (MITZ and UITZ, respectively) and supratidal zones on beaches with cobble, boulder, and pebble cover. Because the Harlequin Duck diet is largely epibenthic, collected from the lower intertidal zone (LITZ) (Robertson and Goudie 1999), the asphaltic residues are very unlikely targets in their diet, and the SOR is so highly weathered that the PAHs are essentially not bioavailable (Integral Consulting 2006; Taylor and Reimer 2008), consumption of SOR is not considered to be a plausible pathway for current PAH exposures to seaducks and therefore was not modeled.

Similarly, the possibility that a Harlequin Duck would directly excavate SSOR at the depth at which it exists under surface-armored sediments in the MITZ and UITZ is extremely unlikely because, as noted above, PWS seaducks feed on epibenthos in the LITZ, and they do not have the physical capability to dig in such sediments to those depths or under such armoring (Robertson and Goudie 1999; Eadie et al. 1995, 2000). Consequently, the pathway in which a seaduck excavated sediments to feed on infauna and in the process intersected SSOR is too implausible to warrant modeling. The pathway in which a seaduck collected prey from a recently excavated sea otter pit that intersected SSOR was also analyzed in Harwell et al. (2012) and shown to be implausible.

However, seaducks are exposed to PAHs in prey tissues, sediments, and seawater in the PWS environment, providing the 3 potential routes of exposure that warranted quantitative assessment here.

The seaduck IBM was parameterized with empirical data for each of these 3 exposure pathways, including: 1) the most recently measured PAH concentrations in PWS prey, sediments, and seawater (data summarized in Harwell et al. [2012], based on samples reported in Boehm et al. [2004, 2007], Neff et al. [2006], and Brannon et al. [2007]), 2) dietary information on PWS Harlequin Ducks (data from Patten et al. [2000] as aggregated in Harwell et al. [2012]), 3) other literature-derived data about seaduck behavior and physiology (Birt-Friesen et al. 1989; Nagy et al. 1999; McKinney and McWilliams 2005; Guillemette et al. 2007), and 4) energy densities of each prey category (Cummins and Wuycheck 1971; Wacasey and Atkinson 1987). Sources for all data are discussed and cited in Harwell et al. (2012), and the PAH concentration data for prey tissues, sediments, and seawater used in the model are available online (http://www.valdezsciences.com/polycyclic_aromatic_hydrocarbon.cfm).

The data used in the model for the environmental concentrations of PAHs in PWS are presented in Table 1. Differences in mean TPAH concentrations between oiled and reference areas may be seen in sediments and some of the prey species, with overlapping standard deviations. Mean seawater TPAH concentrations were very similar between oiled and reference sites, occurring at extremely low levels, measured in parts-per-trillion, consistent with Pope et al. (2011).

**Table 1:** TPAH concentrations in oiled and reference prey, sediments, and seawater

	Oiled			Reference			
			
	Mean	SD	*n*	Mean	SD	*n*	Proportion of PWS seaduck diet^b^ (%)
Prey Tissues TPAH^a^ (ng · g^−1^ dry weight)							

Prey category							

Gastropods	23.04	8.09	26	20.76	8.42	7	59.8

Mussels	45.87	28.99	96	33.11	17.99	27	12.3

Fish eggs	53.07	3.09	12	54.15	3.87	23	12.0

Crustaceans	25.54	4.80	15	31.07	7.85	4	9.6

Clams	72.08	68.88	54	42.69	22.14	12	2.3

Other inverts	107.65	102.43	26	47.82	24.25	6	4.0

Sediments TPAH^a^ (ng · g^−1^)	63.21	132.37	51	28.27	33.27	27	

Seawater TPAH^a^ (ng · L^−1^)	8.48	3.57	23	7.62	1.54	12	

PAH = polycyclic aromatic hydrocarbons; PWS = Prince William Sound; SD = standard deviation; TPAH = total PAH.

a Data and sources reported in Harwell et al. (2012). All data available online at: http://www.valdezsciences.com/polycyclic_aromatic_hydrocarbon.cfm.

b Data from Patten et al. (2000) based on length-importance index; categories aggregated as reported in Harwell et al. (2012).

Closer examination of the PAH distributions in the sediment and prey data shows complex patterns of spatial and PAH-source heterogeneity that are relevant to understanding current risks (AE Bence, AEB Services, LLC, Corpus Christi, TX, personal communication). In general, there are 5 sources of PAHs in PWS that can be identified by the patterns of the relative frequencies of the 40 analyzed PAHs: 1) petrogenic PAHs from natural petroleum background, 2) petrogenic PAHs from EVOS, 3) petrogenic PAHs from diesel fuel, 4) biogenic PAHs, dominated by perylene, and 5) pyrogenic PAHs from local point sources of past industrial activity (Page et al. 1999) or atmospheric deposition of combustion products.

For sediments, all but 1 of the reference-site samples (*n* = 27) and approximately 80% of the oiled-site samples (*n* = 51) were below 60 ng · g^−1^ (ppb) TPAH. Of the 11 oiled sediment samples exceeding 60 ppb TPAH, 6 showed a characteristic EVOS fingerprint, 3 had no EVOS fingerprint but very high levels of perylene, and the other 2 had non-EVOS petrogenic patterns. The 1 elevated reference sample had both elevated perylene and non-EVOS petrogenic patterns. However, as discussed below, the quantitative risk assessment of Harwell et al. (2012) showed clearly that the oiled sediments pose essentially no risk to the seaducks, so whatever detectable EVOS signal exists on top of the background PAHs (found here in only approximately one-tenth of the sediment samples) is insignificant.

Data for the prey species also showed heterogeneous patterns of TPAH concentrations and sources. As might be expected for filter-feeders, the mussel and clam patterns were similar to the sediment pattern. For mussels, 21 (approximately 20%) of the oiled samples (*n* = 96) had TPAH above 40 ppb; of these, 13 had petrogenic sources with an EVOS signal (mixed with substantial fractions of pyrogenic PAHs), 6 were mixtures of pyrogenic and non-EVOS petrogenic PAHs, and 2 were dominated by perylene. The 2 reference mussel samples (*n* = 27) that exceeded 40 ppb contained mixtures of diesel and pyrogenics. For clams, 16 (30%) of the oiled samples (*n* = 54) were at least 50 ppb; of these, 4 were petrogenic with an EVOS signal, 6 were petrogenic with no EVOS signal, and 6 were pyrogenic (mostly mixed with diesel). One of the reference clam samples (*n* = 12) reached 50 ppb, a mixture of non-EVOS petrogenic and pyrogenic PAHs.

On the other hand, the oiled polychaete statistics were driven by a single sample (*n* = 26), which had a very high TPAH concentration (over 450 ppb), showing no EVOS signal but dominated by diesel fuel and pyrogenic sources. Of the other 11 oiled polychaetes exceeding 50 ppb, 5 showed a detectable EVOS signal (mixed with pyrogenics) and 6 were non-EVOS petrogenics mixed with pyrogenics. For the algae-feeding gastropods, which constitute almost 60% of the seaduck diet in PWS (Table 1), none of the 26 oiled samples or 7 reference samples showed any EVOS signals; rather, they were all dominated by combustion products.

Despite this complex pattern of spatial heterogeneity and diversity of sources of PAHs, we modeled the exposures to seaducks with the conservative assumption that all doses to the oiled seaducks were attributable to EVOS, where conservative here means overestimating the exposures. The seaduck IBM was structured similarly to the sea otter IBM, with hourly time steps and with random sampling from the complete distributions of TPAH and of PAH proporations that were derived from the field samples in both oiled and unoiled areas. Average daily assimilated doses for each PAH analyte and for TPAH were calculated for a population of 500 000 seaducks for each of 8 age- and gender-based classes, capturing the expected exposure variability within a population of Harlequin Ducks living in PWS. A series of sensitivity analyses was carried out to explore model uncertainty. As for the sea otters, emphasis was conservatively placed on the 99.9% quantile most-exposed individuals.

The effects component of the risk assessment began with a search of the EcoSSL database, but no studies were found that met the criteria for establishing reliable avian TRVs for PAHs (USARMY 2000; USEPA 2005). However, studies by Stubblefield, Hancock, Ford et al. (1995) and Stubblefield, Hancock, Prince et al. (1995) on Mallard Ducks using weathered EVO uniquely provided a reasonable basis for assigning TRV values that are applicable to potential Harlequin Duck exposures to EVOS-derived PAHs. Those authors measured a suite of endpoints, including the 2 population-relevant parameters used here: eggshell thickness and eggshell strength. Accordingly, Harwell et al. (2012) applied the USEPA protocols (USEPA 2005, 2007) to the Stubblefield, Hancock, Ford et al. (1995) and Stubblefield, Hancock, Prince et al. (1995) studies to derive gender-specific chronic NOAEL and LOAEL TRVs normalized to body weight. Because those experiments involved dosing with weathered *Exxon Valdez* crude oil as a mixture, TRVs could only be derived for the TPAH of the mixture, not for the separate PAH ring groups as was done for sea otters, because the measured toxicity cannot be attributed to individual PAHs or to specific PAH-ring groups. On the other hand, by using weathered EVO as a mixture, the toxicity experiments are directly relevant to the issue at hand, the toxicity and subsequent risk from exposure to weathered EVO-derived PAHs. Consequently, these experiments provide an appropriate basis for establishing avian chronic TRVs for this ecological risk assessment.

## RESULTS AND CONCLUSIONS

By using the individual-based models described here, Harwell, Gentile, Johnson et al. (2010) and Harwell et al. (2012) were able to simulate a large number of individual sea otters or seaducks, thereby generating frequency distributions of assimilated doses. Those distributions became the bases to reach clear conclusions about the risks to individuals living under the current conditions in PWS. We next present a methodology to extrapolate quantitatively from the individual-level distributions to derive estimates of population-level risks. This approach allows risk managers to understand the full spectrum of risks to particular populations of concern in the environment. This more complete understanding, in turn, may better inform the decision-making process.

### Sea otter model results

In the present study, we have added to the sensitivity analyses reported in Harwell, Gentile, Johnson et al. (2010) a new set of simulations to estimate how many SSOR-intersecting pits a sea otter would need to excavate per day to reach a toxicity threshold for individual-level effects. This was done by bypassing all of the probabilistic algorithms of the sea otter IBM for determining if an SSOR-intersecting pit was excavated during a particular time step. In place of these calculations, the model was fixed to create exactly 1 SSOR-intersecting pit every day, keeping the remainder of the stochastic processes in the model intact (e.g., retaining the relative frequency of SSOR categories: 8.3% HOR, 33.3% MOR, 31.7% LOR, and 26.7% oil-film trace residues). Forcing 1 SSOR-intersecting pit per day contrasts with the base model predictions of 1 SSOR-intersecting pit occurring, on average, about once every 50 days for the pup class of sea otters, and up to once per 180 days for adult females without a pup (Table 2).

**Table 2:** Average number of days for 1 sea otter pit to intersect SSOR

Sea otter class	Days
Pup	51

Juvenile	64

Subadult	64

Male nonterritorial	120

Male territorial	120

Female with pup	78

Female without pup	180

SSOR = subsurface oil residues.

Outputs from the 1-pit-per-day series of simulations for the 99.9% quantile sea otter remained well below the NHQ and LHQ levels (Table 3). The assimilated doses from this fixed number of pits-per-day model are linear with increasing number of pits, e.g., 2 pits per day would produce exactly twice the assimilated doses of 1 pit per day. Because of this linearity, we can estimate the number of pits per day necessary to reach an HQ1 = 1 (Table 4); this was done by heuristically increasing the 1-pit-per-day-model outputs by multiplicative factors and quantifying where the HQ1 = 1 threshold is crossed. Thus, for the maximum-exposed sea otters (i.e., the 99.9% quantile individuals), approximately 4 pits per day would reach the 4–6-ring NHQ, and approximately 10 pits per day for the LHQ.

**Table 3:** NOAEL and LOAEL hazard quotients for 1-pit-per-day scenario

	99.9% Quantile NHQ Values			99.9% Quantile LHQ Values		
		
	2–3 Ring	4–6 Ring	TPAH	2–3 Ring	4–6 Ring	TPAH
Pup	0.044	0.266	0.153	0.022	0.104	0.095

Juvenile	0.043	0.251	0.154	0.022	0.098	0.095

Subadult	0.042	0.255	0.152	0.021	0.100	0.094

Male territorial	0.034	0.211	0.125	0.017	0.082	0.077

Male nonterritorial	0.034	0.207	0.123	0.017	0.081	0.076

Female with pup	0.030	0.185	0.108	0.015	0.072	0.067

Female without pup	0.039	0.239	0.143	0.020	0.093	0.088

LHQ = LOAEL hazard quotients; LOAEL = lowest-observed-adverse-effects-level; NHQ = NOAEL hazard quotients; NOAEL = no-observed-adverse-effects-level; TPAH = total polycyclic aromatic hydrocarbons.

**Table 4:** Number of pits per day required to reach NOAEL and LOAEL TRVs for the 99.9% quantile individuals in the base sea otter model

	NOAEL TRV			LOAEL TRV		
		
	2–3 Ring	4–6 Ring	TPAH	2–3 Ring	4–6 Ring	TPAH
Pup	22.9	3.8	6.5	46.0	9.6	10.5

Juvenile	23.1	4.0	6.5	46.4	10.2	10.5

Subadult	23.7	3.9	6.6	47.6	10.0	10.6

Male territorial	29.4	4.7	8.0	58.9	12.2	12.9

Male nonterritorial	29.4	4.8	8.1	59.1	12.4	13.1

Female with pup	32.9	5.4	9.2	66.0	13.9	14.9

Female without pup	25.5	4.2	7.0	51.2	10.7	11.3

LOAEL = lowest-observed-adverse-effects-level; NOAEL = no-observed-adverse-effects-level; TPAH = total polycyclic aromatic hydrocarbons; TRV = toxicity reference values.

The next step was to extend this analysis to assess population-level effects. The rationale for our approach is this: if the 99.9% quantile sea otter did actually reach the TRV levels, i.e., if the sea otter dug 4–10 SSOR-intersecting pits every day, then there still would only be 1 sea otter in 1000 showing effects, clearly a level of incidence that would be neither detectable nor of consequence in a real population. Then the question becomes at what quantile level would an effect become manifest in a population, that is, what percentage of individuals would need to be affected for there to be a detectable and significant change in the population? We reasoned that at the median level (50% quantile, or half the population being affected), effects would likely be detectable in the population. Precisely where between the 50% quantile and the 99.9% quantile a population-level effect would be detectable relates in part to the natural variability of the population. For example, a 10% change in a population with, say, an interannual variability of 10 ×, such as some forage fish populations, would not likely be detectable, whereas a 10% change in a population with virtually no interannual variability, such as some marine mammals, might be. However, rather than our selecting a single quantile level for indicating population-level effects, we generated a family of curves representing several quantile levels, ranging from the 50% quantile to the 99.9% quantile.

**Figure 3:**
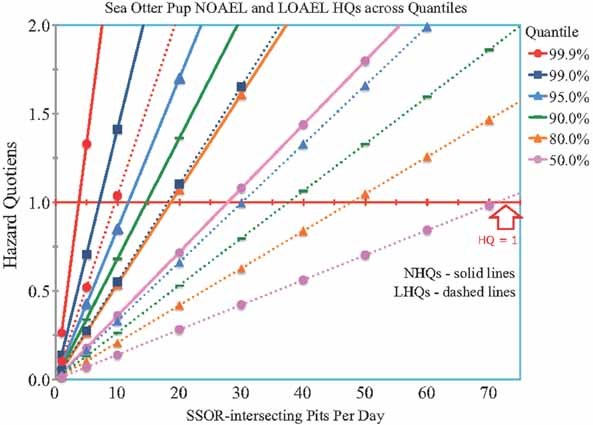
Hazard quotients (HQs) for pup class of sea otters across quantiles for the 4–6-ring PAH toxicity reference values (TRVs). Quantiles presented based on sea otter model outputs for hypothetical multiples of the 1-SSOR-intersecting-pit-per-day scenario (see text for discussion; for context, note that actual incidence estimated as a single sea otter pit intersecting SSOR about once every 50–180 days, depending on the class of sea otter). Results based on simulations of a population of 500 000 individuals. Solid lines represent HQs for the no-observed-adverse-effects (NOAEL) TRV; dashed lines represent HQs for the lowest-observed-adverse-effects (LOAEL) TRVs. Horizontal red line represents TRV threshold (i.e., HQ = 1). From these families of curves, the risk manager can chose bounds of expected population-level effects.

As an example, Figure 3 shows the NOAEL and LOAEL hazard quotients (NHQ = 1 and LHQ = 1, respectively) for various quantiles for assimilated doses of 4–6-ring PAHs by the pup class of sea otters over a heuristic range of SSOR-intersecting pits per day. This family of curves illustrates the consequences of selecting alternate quantile levels, as well as the choice of NOAEL- or LOAEL-based thresholds. For instance, the relationship between the number of SSOR-intersecting pits per day and resulting NOAEL-based HQs for the median level (50% quantile) exposure of the simulated population is below the similar relationships for the SSOR-intersecting pits per day and LOAEL-based HQs for all quantiles up to the 95% level.

**Figure 4:**
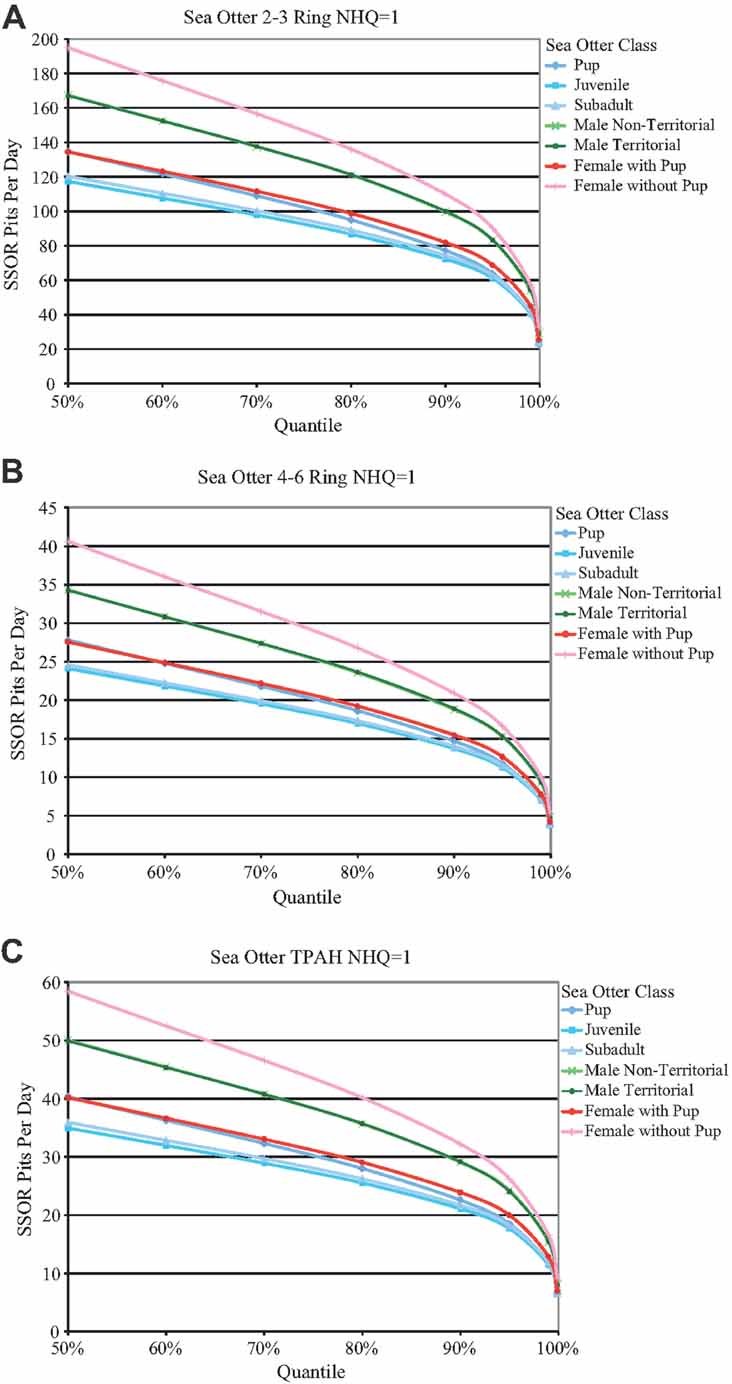
NOAEL hazard quotients (NHQs) across quantiles for 7 classes of sea otters. Quantiles based on model simulations of a population of 500 000 individual sea otters for hypothetical multiples of the 1-SSOR-intersecting-pit-per-day scenario. (A) HQs for the 2–3-ring PAHs TRV. (B) HQs for the 4–6-ring PAHs TRV. (C) HQs for the TPAH TRV.

**Figure 5:**
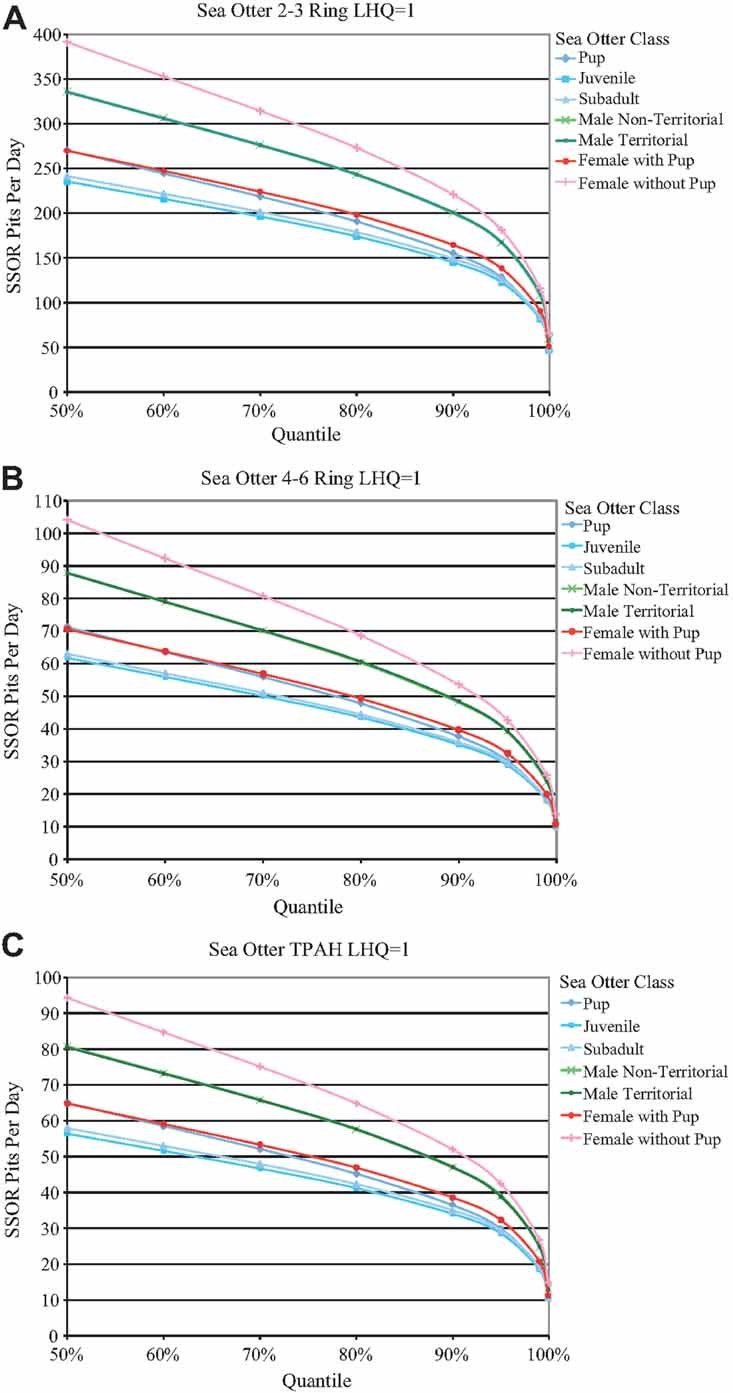
LOAEL hazard quotients (LHQs) across quantiles for 7 classes of sea otters. Quantiles based on model simulations of a population of 500 000 individual sea otters for hypothetical multiples of the 1-SSOR-intersecting-pit-per-day scenario. (A) HQs for the 2–3-ring PAHs TRV. (B) HQs for the 4–6-ring PAHs TRV. (C) HQs for the TPAH TRV.

The effects of sea otter age-gender class and of selecting a particular quantile level become clear when the outputs from the same sensitivity analysis are plotted to assess at what frequency of SSOR-intersecting pits would the NOAEL- and LOAEL-based thresholds be crossed (i.e., NHQ = 1 and LHQ = 1, respectively) for the 2–3-ring, 4–6-ring, and TPAH TRVs across the 7 classes of sea otters (Figure 4 and Figure 5, respectively). For instance, these figures show that the 4–6-ring PAH exposures have the highest risk across all sea otter classes for reaching NOAEL levels, whereas the 4–6-ring and TPAH exposures have about the same risks for reaching the LOAEL levels (where higher risk here means fewer SSOR-intersecting pits per day are needed to reach the HQ effects thresholds). These figures also indicate that: 1) the pup class of sea otter is almost twice as at-risk as the adult female without pup class, 2) the nonadult sea otter classes cluster together as being similarly sensitive, 3) there is no difference between the adult males in the territorial and nonterritorial phases, but 4) the adult female with pup class is almost as sensitive as the nonadult classes and is quite different from the adult female without a pup. The shape of these curves also illustrates that for each class of sea otter, the quantile level of reaching effects thresholds is not a sensitive factor (<2 ×) and is essentially linear over the range of 50%–90% quantiles, but is quite sensitive and nonlinear above 90%; however, the latter region applies only to the infrequent, higher-exposed individuals and therefore is not relevant to detectable population-level effects.

**Figure 6:**
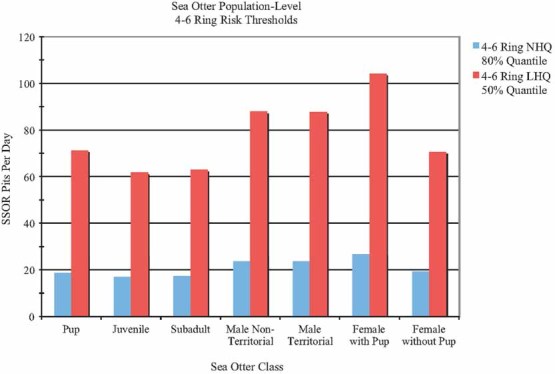
Thresholds for detecting population-level effects from exposures to 4–6-ring PAHs by 7 classes of sea otters. Suggested bounds of detecting population-level effects are: no population-level effects would be detectable at exposures below the NHQ 80% quantile (i.e., ≤20% of individuals reaching NOAEL exposures) and population-level effects are expected to be detected at exposures above the LHQ 50% quantile (i.e., ≥50% of individual reaching effects levels). Other bounds could be chosen by the risk manager.

Finally, the population-level risk picture that emerges with particular assumptions about risk thresholds is illustrated in Figure 6. Here we assumed that no population-level effect would be detectable for exposures in which 20% or less of the population reached the NOAEL (i.e., below the 80% quantile NOAEL HQ level); this is quite conservative, because even if 100% reached the NOAEL levels, there supposedly, by definition, would be “no effects.” Conversely, we assume that detectable population-level effects could be expected where half or more of the population exceeded the LOAEL-based HQ threshold (i.e., 50% quantile LOAEL HQ level). Thus, this figure shows that across all sea otter classes, anything below approximately 18–25 SSOR-intersecting pits per day would not result in a detectable population-level effect, whereas anything above approximately 60–100 SSOR-intersecting pits per day (depending on the class of sea otter) would likely be detectable as a population-level effect. One might choose different quantile levels, but in general this approach provides a range of values for the risk manager to consider and to put into the context of existing risks. For the case here, the projected values necessary to cause detectable population-level effects contrast considerably with the actual effective rate in the PWS ecosystem, estimated as a single sea otter pit intersecting SSOR about once every 50–180 days, again depending on the class of sea otter.

This method in essence allows a quantitative understanding of population-level characteristics to emerge from the distributions of outputs from an individual-based model. Using this approach, either for a case in which the actual environmental exposures are in the range of toxicity thresholds or for a case, such as here, where the environmental exposures have to be heuristically increased to approach toxicity thresholds, a risk manager would be able to select the level of detectability that seems appropriate for the endpoint and/or VEC at-hand and then make a determination using the appropriate model output curve. Furthermore, reporting the range of values across quantiles, similar to considering the range of values across the NOAEL- and LOAEL-based TRVs, can provide risk managers with a comparative context for assessing the magnitude of the risks, the potential for individual-level effects, and, importantly, the potential for population-level effects.

### Harlequin Duck model results

Harwell et al. (2012) showed that the assimilated doses to the maximum-exposed individual Harlequin Ducks are approximately 400–4000 times lower than the toxicity reference values (NOAEL and LOAEL, respectively) established using USEPA protocols for chronic exposures of PAHs. These authors concluded that these exposures are so low that no individual-level effects are plausible, even within a simulated population that is orders-of-magnitude larger than exists in PWS.

The cumulative assimilated doses in the seaduck simulations provide a more comprehensive picture of the sources of PAHs in PWS, with the seaducks acting as an integrator across the environmental variability in PAH sources and spatial heterogeneity. Harwell et al. (2012) showed that the sediment-based exposure pathway (which constituted approximately 10% of the overall assimilated dose) did show a detectable EVOS signal, whereas the prey-based pathways (which constituted approximately 90% of the total dose) showed no such EVOS signal, and the reference and oiled PAH distributions were essentially the same. Thus, the EVOS signal that was detectable in approximately one-tenth of the sediment samples was also detectable in the seaduck assimilated doses, but only at levels that could cause no effects on any seaducks. The similarly infrequent EVOS signal in some of the prey samples, however, was lost in the assimilated doses to seaducks for 2 reasons: 1) the significant contributions of other non-EVOS sources to the prey PAHs, as discussed previously, and 2) the significant influence of the method detection limits (MDLs), which were reached in approximately 75% of the analyzed PAHs in both oiled and reference prey samples because their concentrations were so low, as discussed more fully in Harwell et al. (2012).

From the PAH distributions in the sediment and prey samples and the resultant PAH distributions in the seaducks' assimilated doses, the pattern that emerges is: 1) the concentrations of residual EVOS-derived PAH in the PWS environment are extremely low and constitute no significant risk to any individual seaduck, 2) background PAH levels are spatially heterogeneous and derive from variable mixtures of natural petrogenic background, diesel, biogenic, and pyrogenic sources, 3) there remains a detectable EVOS signal in some sediments in oiled areas, with a spatial and PAH-source heterogeneity of about a 10-to-1 split between samples that had completely returned to background versus samples with a residual EVOS signal that, although detectable, poses no risk, and 4) there is no detectable EVOS signal in the assimilated doses to seaducks from the prey pathways.

Harwell et al. (2012) next asked the question, how much would the environmental concentrations have to be in PWS in order for there to be individual-level effects? To address this question, the authors artificially increased the environmental concentrations of TPAH heuristically, bracketing the HQ = 1 thresholds. This was done by modifying the Harlequin Duck model so that a multiplier was applied to each PAH concentration selected for each pathway during each model time step. Using this approach, the relative proportional contributions from the various PAH sources to the assimilation pathways of the seaduck were preserved. The range of multiplier values that were applied in the heuristic analyses was derived from linear extrapolations of the base model results.

**Figure 7:**
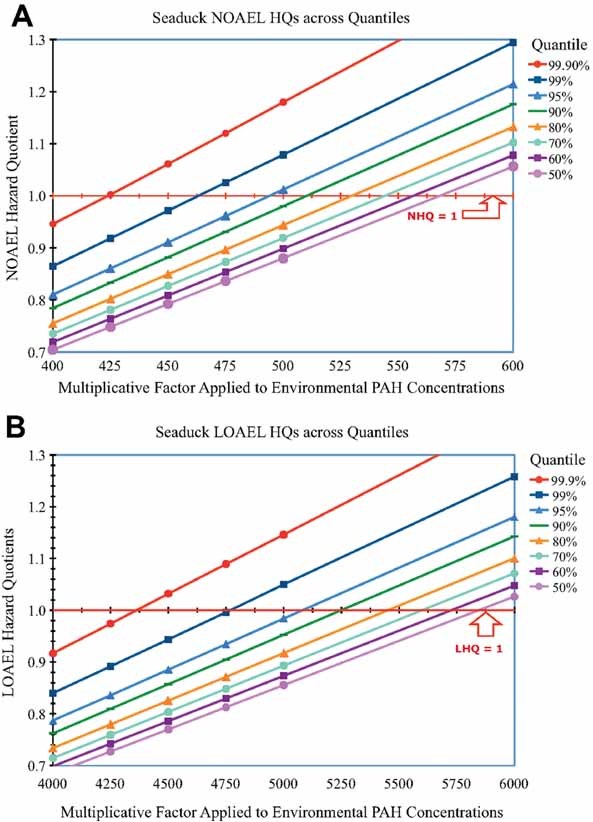
Hazard quotients across quantiles for seaducks. Quantiles based on model simulations of a population of 500 000 individual seaducks exposed to hypothetical multiples of the most-recent data for actual PAH concentrations in the Prince William Sound environment (see text for details). Hazard quotients based on 4–6-ring PAH TRVs. Horizontal red line represents TRV threshold (i.e., HQ = 1). (A) NOAEL HQs. (B) LOAEL HQs. Modified by forthcoming permission of Taylor & Francis (http://www.tandf.co.uk/journals) from Harwell et al. (2012).

The results of this series of simulations are shown in Figure 7, reproduced from Harwell et al. (2012). The Harlequin Duck category of adult female in winter is reported here; all of the other seaduck categories had very similar results. Note that the family of quantile curves cluster more closely than the comparable curves from the sea otter model (Figure 3). This is because the dominant factor causing the higher-level exposures to sea otters was the intersecting of 1 or more sea otter pits into a patch of SSOR during the simulated day (a rare but high-consequence event), a pathway that is not possible for seaducks. The variability in seaducks, on the other hand, is driven solely by the lognormal distributions of PAH concentrations in the various sources, i.e., the diet categories, sediments, and seawater. Another factor to note in the seaduck results is the large difference between the NOAEL and LOAEL families of hazard quotient curves. This results from the use of a single toxicity test to derive the TRVs and from the particular experimental concentrations applied in those tests, in contrast with the sea otter TRVs, which were derived statistically from over 40 toxicity studies.

**Figure 8:**
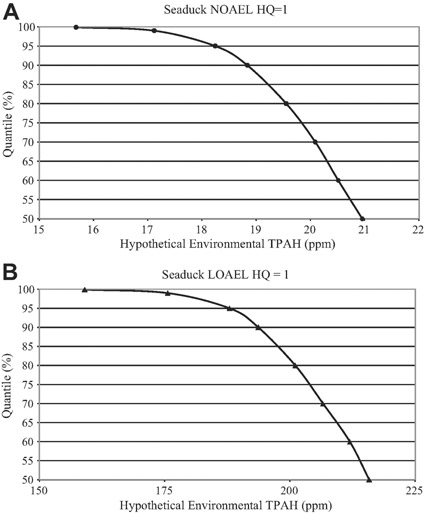
Hypothetical environmental PAH concentrations needed for seaducks to reach HQ = 1 across quantiles. Quantiles based on model simulations of a population of 500 000 individual seaducks exposed to hypothetical multiples of the most-recent data for actual PAH concentrations in the Prince William Sound environment. Hazard quotients based on 4–6-ring PAH TRVs. (A) NOAEL HQ = 1. (B) LOAEL H Q = 1. Reprinted by forthcoming permission of Taylor & Francis (http://www.tandf.co.uk/journals) from Harwell et al. (2012).

Figure 8 reflects the hypothetical environmental TPAH concentrations needed to reach the HQ = 1 thresholds for NOAEL and LOAEL, respectively. The environmental TPAH concentrations were calculated by multiplying the weighted mean environmental TPAH concentration (based on proportional contribution to the assimilated dose) by the multiplier factors discussed previously.

Harwell et al. (2012) concluded that the weighted mean environmental TPAH concentrations would have to be approximately 20 ppm to reach NHQ = 1 and 200 ppm to reach LHQ = 1 levels, values not seen in PWS since 1990, an order of magnitude greater than those measured since the cleanup operations were completed in 1991, and 3 orders of magnitude greater than currently exist in PWS. These results reinforced the conclusion of essentially no risk to seaducks remaining from EVOS at present.

**Figure 9:**
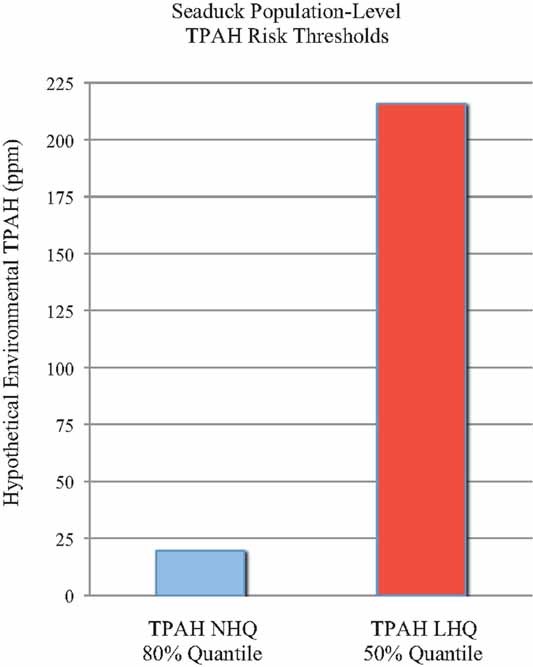
Thresholds for detecting population-level effects from exposures to TPAH by seaducks. Suggested bounds of detecting population-level effects are: no population-level effects would be detectable at exposures below the NHQ 80% quantile (i.e., ≤20% of individuals reaching NOAEL exposures) and population-level effects are expected to be detected at exposures above the LHQ 50% quantile (i.e., ≥50% of individual reaching effects levels). Other bounds could be chosen by the risk manager.

To derive estimates of population-level risks, we used the same reasoning for the seaduck as previously discussed for the sea otters: we assumed that below the environmental concentrations of TPAH resulting in an assimilated dose that reaches the NOAEL TRV threshold for the 80% quantile (i.e., only 20% of the individuals in the population assimilating doses that exceed the NHQ = 1 threshold), no population-level effects would be detectable, whereas any environmental TPAH concentrations that resulted in doses that exceed the LOAEL TRV threshold for the median-exposed (50% quantile) seaduck likely would be detectable in the population. Consequently, Figure 9 shows the ranges of values within which population-level effects would first be detectable. Again, the risk manager may choose alternate thresholds for inferring detectable population-level effects, but as Figure 8 shows, the selection of a particular set of thresholds is not a sensitive parameter (e.g., using a 80% quantile LHQ value instead of the 50% quantile value results in the expected population-level effects being detectable at approximately 200 ppm rather than approximately 215 ppm).

To put these environmental TPAH concentrations in perspective, Harwell et al. (2012) examined the most relevant data of PAH measurements in PWS soon after the EVOS. These data were collected for: 1) the Natural Resource Damage Assessment (NRDA) conducted by NOAA, the US Departments of Interior and Agriculture, and the State of Alaska Departments of Law, Fish & Game, and Environmental Conservation, labeled PWSOIL (see the *Exxon Valdez* Trustees Database [EVTHD] reported in Short and Nelson [1998]), and 2) the ad hoc Oil Spill Health Task Force (OSHTF) assessments conducted by scientists from federal, state, and local agencies and from Exxon (cited in Bence and Burns [1995]). The PWSOIL data showed that TPAH in the range of 10–40 ppm (dry weight basis) was found in only 2% of sampled mussels in the first few weeks after the spill, none were found that exceeded 10 ppm beyond 90 days after the oil spill, and none exceeded 1 ppm after 1990; the pattern for clams was very similar. The OSHTF data, which were on a wet-weight basis only, were consistent with PWSOIL, with 92% of mussels in 1989 below 1 ppm (approximately 10 ppm dry weight, assuming a 90% water content), and none exceeding that level after 1990; that database also had no clams exceeding 1 ppm (wet weight) at any time following the oil spill. Furthermore, consider that the model results are based on mean environmental TPAH concentrations, requiring a high fraction of the diet to have the estimated concentrations, not just a few clams or mussels.

Based on these data and the results of the HQ = 1 sensitivity analyses, we can conclude that even as early as 90 days after EVOS, the mean measured environmental TPAH levels in PWS were well below the Harlequin Duck population-level-effects thresholds estimated through the heuristic sensitivity analyses. Consequently, the prospects for population-level effects occurring now, more than 20 years after the oil spill, are essentially nonexistent.

### Using IBMs for population-level assessments

As we have seen, properly constructed IBMs that are designed to address ecotoxicological risk assessment issues can provide useful insights into population-level risks in a manner and with an efficacy that exceeds traditional population models. In part, this derives from the relative ease of constructing IBMs: The availability of a comprehensive object-oriented simulation and user-interface software such as *Stella*™ makes this type of modeling accessible to many ecologists who might otherwise be intimidated by the mathematical or programming demands of more traditional population-level models. In essence, the IBM construct provides a direct linkage between the ecologists' expertise and the quantitative projection of the implications of that expertise and associated empirical information. More importantly, the IBM approach is much more reflective of what actually unfolds in the environment, particularly as it relates to potential cause-effects relationships on individuals and collectively on populations.

The risk-based conceptual models, illustrated here, can be derived directly from the ecological understanding of how the system operates at the level of individuals (i.e., typically the level at which most relevant data are acquired), rather than being founded on and significantly limited by mathematical abstractions of population-level dynamics, for which often there are few data and significant constraints on realistically simulating complex relationships and events.

Quantitative IBMs can directly translate such a conceptual model into a quantitative tool for readily characterizing risk. Additionally, because of its flexibility and realistic construct, IBMs are readily amenable to conducting a suite of relevant experiments to fully explore the entire risk regime. For example, Harwell, Gentile, Johnson et al. (2010) used sensitivity analyses to quantify the effects of changing input data by comparing the sea otter risks from 2 different databases on sea otter diving characteristics: the Garshelis and Johnson (2001) observational database discussed previously and the projected risks using the Ballachey and Bodkin (2006) database. Similarly, Harwell, Gentile, Johnson et al. (2010) conducted a series of structural sensitivity analyses to examine alternate model constructs using different approaches to assessing co-occurrence; for example, 1 set of sensitivity analyses contrasted the detailed spatial and behavioral data-driven approach of the Harwell, Gentile, Johnson et al. (2010) base model with the more simplistic approach to calculating SSOR-intercepting probabilities of Short et al. (2006), in which, for example, sea otters were incorrectly assumed to forage throughout all tidal zones and in all sediment habitats.

Sensitivity analyses are readily conducted using IBMs to examine the magnitude and sources of uncertainty and the relevance of various types and sources of uncertainty for the conclusions that are derived from the model simulations. IBMs may also explicitly incorporate spatial and temporal heterogeneity as measured in the environment or, conversely, may be used to examine the importance of alternative patterns of heterogeneity and variability. Interactions among stressors, implications of different thresholds, alternative pathways of exposures, and other relevant factors potentially affecting risk may be explored. IBMs can be data-intensive, which may limit their use to the larger-scale or more complex ecological risk assessments. However, other than characterizing the exposure regime, which is by necessity a site-specific data requirement, often much of the needed data for an IBM may be derived from the literature, as was done for the 2 IBMs discussed here. Typically, in any large-scale NRDA or oil spill situation, extensive data collection efforts are conducted after the event to establish injury, characterize variability, and specify recovery goals. Thus, if the development of an appropriate toxicological risk assessment model is identified as a goal at the beginning of the experimental design process for data collection, then the types of site-specific data needed for a comprehensive risk assessment, such as illustrated here, can be forthcoming, bringing the valuable tool of IBMs to bear on the risk assessment and management problems. Additionally, the ease of conducting sensitivity analyses means that the implications of having limited or uncertain data may be directly tested and evaluated. Moreover, a library of IBMs could be developed that could be drawn on and adapted to smaller-scale environmental problems. Recently there have been calls for enhanced use of modeling for ecological risk assessments in support of more routine regulatory decisions, such as registering pesticides in Europe (Forbes et al. 2009).

An IBM also may be used to derive emergent attributes of the system. This capability was demonstrated in the evaluation of the net proportions of PAH analytes in the environment (Harwell et al. 2012): by treating the model-simulated individuals as integrators of all the assimilated dose pathways from all the environmental sources of PAHs in proportion to their actual contributions to real seaducks, the IBM became a unique and invaluable diagnostic tool to identify the origins of the environmental PAHs (i.e., whether from pyrogenic or petrogenic sources, and in the latter case, whether from EVO or from some other petroleum sources).

An IBM as described here for individual- and population-level risk assessments has certain specific needs, especially a stochastic structure that allows the capture and analysis of variability and uncertainty. For instance, deriving the quantiles of risks as presented here required a sufficiently large number of individual simulations so that the spatial heterogeneity in the environment and the vagaries of individual actions could be adequately characterized. The was accomplished using the built-in functions in *Stella*™ for generating random numbers, but similar algorithms could be created in another computer programming language. These algorithms actually generate pseudorandom numbers, in that once the algorithm is entered with a specific seed, then the sequence of numbers subsequently generated is deterministic. Our model quality assurance (QA) plan required that every single simulation be reproducible. Thus, the set of simulations done in a particular session had to begin with a user-specified seed rather than one assigned randomly. (The latter case is used when reproducibility is not needed, and the seed is often internally generated at random, such as deriving a seed from the last few digits of the highly precise time of day at the initiation of the simulations session.)

Conducting reproducible simulations provides the ability to compare different situations directly (e.g., oiled versus unoiled, using 2 different values for a particular parameter, etc.) by examining paired individual simulations. This reproducible approach also aids in model testing and development, allows others to generate identical simulations to verify results, and standardizes QA simulations to ensure no change has occurred in the model across the millions of simulations other than the desired changes for each experiment. A potential issue with this approach, however, is that when there is such a high number of calls in the model for random numbers, subtle errors may develop that undermine the desired randomness. For instance, even though statistical analyses on early versions of the sea otter IBM (Harwell, Gentile, Johnson et al. 2010) demonstrated that the parameters being selected were random, a pattern was detected during model development in the timing of the occurrence of very low frequency events across independent parameters. Once identified, this problem was readily corrected by incorporating time lags and other modeling measures that decoupled the various random-number generators. Consequently, when the focus is on very low-frequency outcomes (such as in our 99.9% quantile analyses or where there are very rare but high-consequence events like the co-occurrence of a sea otter pit with a patch of SSOR), extra care is required to ensure genuine randomness.

Other practical issues in using IBMs for risk assessments included developing an approach to convert the daily assimilated doses of an individual sea otter or seaduck into average daily assimilated doses across the population. This was needed to compare outputs with TRV values, which are in units of average daily exposures, because the risks being assessed were for chronic, not acute, exposures. The approach, detailed in Harwell, Gentile, Johnson et al. (2010) and Harwell et al. (2012), involved using the IBM to generate primary distributions of daily doses from which secondary distributions were generated from a large number of repeated random samplings of a specified number of days. Similarly, a technique was needed to ensure that a sufficient number of simulations were conducted to capture the desired variability. This was accomplished by deriving several sets of secondary distributions and increasing the number of samplings until the coefficients of variation were below a specified threshold. Other practical techniques will continue to be developed as IBMs are increasingly used for population-level risk assessments.

Ecological risk assessments have largely focused on individual-level risks, often extrapolated from laboratory toxicity studies. Only recently has there been significant, albeit still limited, use of modeling to address population-level issues, as highlighted in a recent special session at the annual SETAC meeting (SETAC 2010). The modeling that has been done has primarily used traditional population-level models, and individual-based models appear to be rarely used, particularly in the United States. Based in part on discussions with leading practitioners in the field (LW Barnthouse, LWB Environmental Services, Inc., Hamilton, OH; SM Bartell, Cardno ENTRIX, Maryville, TN; WP Cropper Jr, University of Florida School of Forest Resources and Conservation, Gainesville, FL; DL De Angelis, U. S. Geological Survey and University of Miami, Coral Gables, FL; WR Munns Jr, US Environmental Protection Agency, National Health and Environmental Effects Research Laboratory, Narragansett, RI; GW Suter II, US Environmental Protection Agency, National Center for Environmental Assessment, Cincinnati, OH; DA Weinstein, Cornell University Department of Natural Resources, Ithaca, NY, personal communications), we believe there is a great potential for using IBMs in particular to support population-level ecological risk assessments, and that apparent impediments to such usage, such as a lack of appreciation of the potential use of IBMs, extensive data needs or other knowledge limitations, unnecessary intimidation by mathematical or modeling requirements, misperception by funding agencies of the time and funding commitments needed, or other factors, should and can be overcome. The limitations of modeling are often emphasized (e.g., the often-heard homily “All models are wrong; some are useful.”), but in the context of ecological risk assessments, precisely the same statement can be made about laboratory or field studies.

The ease of development, flexibility, and utility of individual-based models suggest the time has come for their wider use as tools for understanding ecological risks or potential risks to populations. Graphical representations of model outputs can foster improved understanding of risks and ease communications among scientists, risk managers, and other interested parties. Finally, the methodology presented here can be particularly useful to decision makers, providing a richer suite of risk characterizations that can be explicitly considered in concert with many other factors, such as economic, policy, or societal values, that often are germane to environmental decisions.
